# Patient-reported symptoms and changes up to 1 year after meniscal surgery: An observational cohort study of 641 adult patients with a meniscal tear

**DOI:** 10.1080/17453674.2018.1447281

**Published:** 2018-03-05

**Authors:** Søren T Skou, Kenneth Pihl, Nis Nissen, Uffe Jørgensen, Jonas Bloch Thorlund

**Affiliations:** 1 Research Unit for Musculoskeletal Function and Physiotherapy, Department of Sports Science and Clinical Biomechanics, University of Southern Denmark, Odense; 2 Department of Physiotherapy and Occupational Therapy, Naestved-Slagelse-Ringsted Hospitals, Denmark, Region Zealand, Slagelse; 3 Department of Orthopaedic Surgery, Lillebaelt Hospital in Kolding, Kolding; 4 Department of Orthopaedics and Traumatology, Odense University Hospital, Denmark

## Abstract

**Background and purpose:**

Detailed information on the symptoms and limitations that patients with meniscal tears experience is lacking. This study was undertaken to map the most prevalent self-reported symptoms and functional limitations among patients undergoing arthroscopic meniscal surgery and investigate which symptoms and limitations had improved most at 1 year after surgery.

**Patients and methods:**

Patients aged 18–76 years from the Knee Arthroscopy Cohort Southern Denmark (KACS) undergoing arthroscopic meniscal surgery were included in this analysis of individual subscale items from the Knee Injury and Osteoarthritis Outcome Score and 1 question on knee stability. Severity of each item was scored as none, mild, moderate, severe, or extreme. Improvements were evaluated using Wilcoxon’s signed-rank test and effect size (ES).

**Results:**

The most common symptoms were knee grinding and clicking, knee pain in general, pain when twisting and bending the knee and climbing stairs (88–98%), while the most common functional limitations were difficulty bending to the floor, squatting, twisting, kneeling, and knee awareness (97–99%). Knee pain in general and knee awareness improved most 1 year after meniscal surgery (ES –0.47 and –0.45; p < 0.001), while knee instability and general knee difficulties improved least (ES 0.10 and –0.08; p < 0.006).

**Interpretation:**

Adults undergoing surgery for a meniscal tear commonly report clinical symptoms and functional limitations related to their daily activities. Moderate improvements were observed in some symptoms and functional limitations and small to no improvement in others at 1 year after surgery. These findings can assist the clinical discussion of symptoms, treatments, and patients’ expectations.

Meniscal tears are a common knee injury in adults, with an annual incidence of up to 172 injuries per 100,000 persons (Peat et al. 2014).

Symptoms such as knee pain and mechanical symptoms (i.e., clicking, locking, or catching) are often considered to be related to meniscal tears (Niu et al. 2011, Yan et al. 2011). However, more detailed information on the clinical symptoms and functional limitations that these patients experience is lacking. Although meniscal surgery is not recommended in most patients with a degenerative meniscal tear (Siemieniuk et al. 2017), no consensus exists on when meniscal surgery is in fact indicated (Lyman et al. 2012). As such, an overview of the specific clinical symptoms and functional limitations that improve most following meniscal surgery would be helpful for clinicians and patients in a shared decision-making process discussing benefits, harms, and patients’ expectations of meniscal surgery.

Importantly, meniscal tears often differ with regard to tear type and symptom onset (i.e., traumatic vs. slowly evolving) between younger and middle-aged to older patients (Poehling et al. 1990, Englund et al. 2008, Bergkvist et al. 2016). Hence, symptom patterns and improvements following meniscal surgery may also differ.

The aim of this exploratory study was to determine the most common clinical symptoms and functional limitations and their severity as reported by patients with a meniscal tear undergoing meniscal surgery and in 2 subgroups based on age (40 years or younger and older than 40 years). Furthermore, we investigated which symptoms and functional limitations had improved most at 1 year after meniscal surgery.

## Patients and methods

We followed the STROBE guideline to report this observational cohort study.

### Patients

Participants from the Knee Arthroscopy Cohort Southern Denmark (KACS) cohort were included in this study (Thorlund et al. 2013). KACS is a prospective cohort following adults undergoing arthroscopy for meniscal tears. Participants were consecutively recruited from 4 public hospitals in Denmark between February 1, 2013 and January 31, 2014, and at 1 of the original four hospitals from February 1, 2014 to January 31, 2015.

### Eligibility criteria

In KACS, patients of at least 18 years of age referred for knee arthroscopy by an orthopedic surgeon on suspicion of a meniscal tear (based on clinical examination, injury history, and MRI if considered necessary) were included if they were able to read and understand Danish, had an email address and did not fulfill any of the following exclusion criteria: no meniscal tear at the later surgery; previous or planned reconstruction surgery of the anterior or posterior cruciate ligament in either knee; fractures to the lower extremities within the last 6 months; or inability to reply to questionnaires because of mental impairment (Thorlund et al. 2013). For the present study, all patients with baseline assessment were included. For the analysis of change from baseline to follow-up, only patients with both baseline assessment and 12-month follow-up data were included.

### Outcomes and other variables

Patient characteristics and outcomes were collected using online questionnaires before surgery (median 7 days, interquartile range 3–10 days) and 3 and 12 months after arthroscopic meniscal surgery.

#### Patient characteristics

Patient characteristics included age, sex, height, weight, symptom onset, and duration of symptoms (Thorlund et al. 2013). Symptom onset was assessed by the question: “How did the knee pain/problems for which you are now having surgery develop?” with 3 response options: “The pain/problems have slowly developed over time,” “As a result of a less severe incident (i.e., kneeling, sliding, and/or twisting of the knee or the like),” and “As a result of a severe incident (i.e., during sports, a crash, or a collision or the like).” Duration of symptoms was assessed by the questions: “How long have you had your knee pain/knee problems for which you are now having surgery?” with response options ranging from “0–3 months” to “more than 24 months”.

#### Outcomes

*KOOS.* The Knee Injury and Osteoarthritis Outcome Score (KOOS) is a validated (Roos et al. 1998, Collins et al. 2016) and often used patient-reported outcome in studies concerning patients with meniscal tears (Herrlin et al. 2007, Katz et al. 2013). It consists of 5 subscales (i.e., Pain, Symptoms, Function in daily living (ADL), Function in sport and recreation (Sport/Rec), and knee related Quality of life (QoL)) constituted of several single-item questions with 5 response categories (0–4; typically ranging from “None” to “Extreme” symptoms) concerning specific perceived clinical symptoms, functional limitations, and quality of life (Roos et al. 1998). Hence, it is a possible source of detailed information on specific knee symptoms and functional limitations that patients with meniscal tears experience.

*Knee stability.* As KOOS does not include specific items on knee instability and giving way, the following question was included: “In the last month, have you felt that your knee was unstable or about to buckle?” The patients responded on a 6-point Likert-like scale (0–5) ranging from “Never” to “All the time.” The question was adapted from the Oxford Knee Score (Dawson et al. 1998).

### Statistics

The single-item outcomes were divided into clinical symptoms, defined as Pain and Symptoms subscales items from the KOOS and the knee instability question, and functional limitations and quality of life, defined as the individual items from the other three subscales of KOOS.

Prevalence of clinical symptoms and functional limitations and quality of life were reported as the actual numbers (proportions) of the full study sample. Presence of symptoms was defined as response options 1–4 (leaving out the response option 0, corresponding to no symptoms) on KOOS and response options 1–5 (leaving out the response option 0, corresponding to no symptoms) on the stability question. Severity of symptoms is presented as the actual number of patients with that symptom severity (proportion). Only patients reporting having symptoms (responded 1–4 or 1–5 on the items described above) were included in this analysis. All outcome items were included in the analysis; however, to increase the readability of the manuscript only the 5 clinical symptoms and the 5 functional limitations and quality-of-life items with the highest prevalence were included in the results section, whereas the rest are presented in the Supplementary material. Subgroup analyses based on age (40 years or younger and older than 40 years) were conducted.

We assessed the differences in single-item scores from baseline to 12 months’ follow-up by presenting the distribution of answers at baseline and 12 months for patients with complete data. Only the 5 clinical symptoms and functional limitations and quality-of-life items with the highest effect size are presented in the results, whereas the rest are presented in the Supplementary material. We made subgroup analyses of patients 40 years or younger and patients older than 40 years of age. The comparison was done using Wilcoxon’s signed-rank test. Effect sizes were calculated by the formula r = Z/√N, with Z being the z statistic output of the Wilcoxon signed-rank test and N being the number of observations. The distribution of answers at baseline and 12 months are presented alongside the effect sizes to allow for a comparison of how each item changed from baseline to 12 months follow-up.

Due to the exploratory nature of this study, the significance level was set at p < 0.05, and all analyses were performed in IBM SPSS Statistics (Version 24, IBM Corporation, Armonk, NY, USA).

### Ethics, registration, funding, and potential conflicts of interest

Written informed consent was obtained from all participants. The regional scientific ethics committee of Southern Denmark waived the need for ethical approval (Thorlund et al. 2013).

The observational KACS cohort was pre-registered at ClinicalTrials.gov (NCT01871272).

This study was funded by an individual postdoctoral grant (JBT) from the Danish Council for Independent Research and funds from the Region of Southern Denmark. The funder had no role in any part of the study other than to provide funding. The authors report no conflict of interest related to this study.

## Results

**Figure 1.**
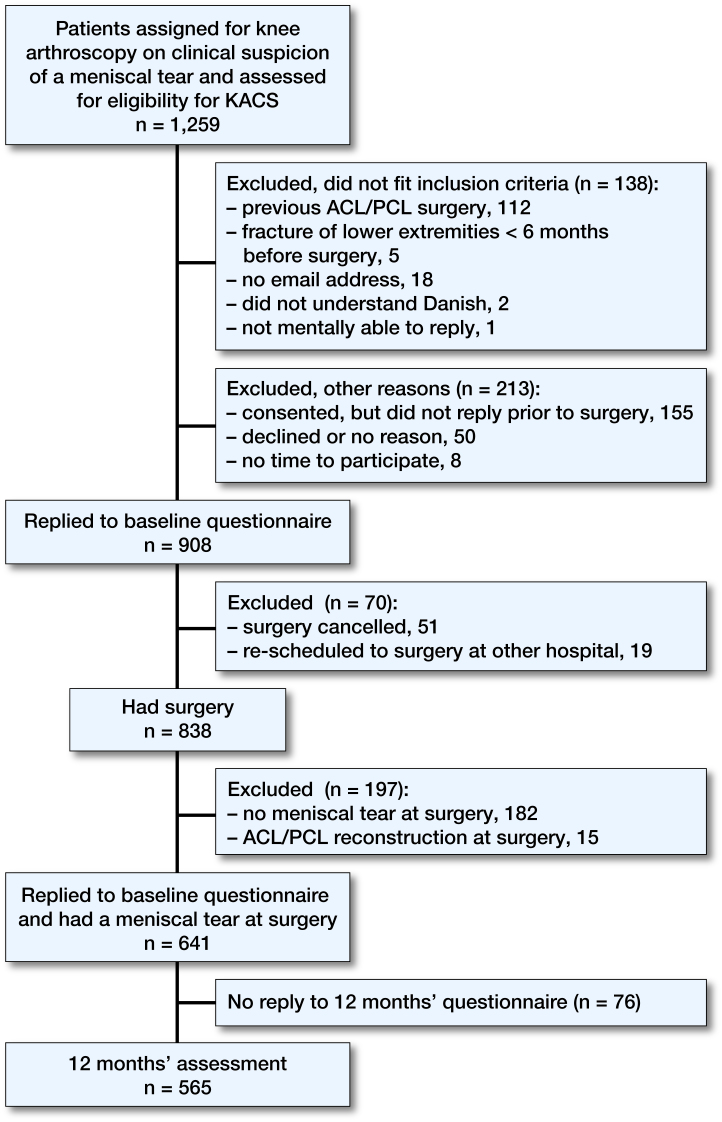
Study flow.

Baseline characteristics for the full group (n = 641) and the subgroups of patients based on age are presented in Table 1, while study flow is presented in Figure 1. 76 (12%) patients failed to reply to the 12 months questionnaire. Of those with complete follow-up, 60 (11%) patients self-reported additional surgery of their knee during the follow-up period.

**Table 1.** Patient characteristics at baseline

	All	≤ 40 years	> 40 years
Variables	(n = 641)	(n = 150)	(n = 491)
Age, years (SD)	49 (13.0)	31 (7.2)	55 (8.6)
Female, n (%)	280 (44)	50 (33)	230 (47)
BMI (SD)	27 (4.4)	26 (4.2)	28 (4.5)
Symptom onset, n (%)			
Developed slowly over time	208 (32)	29 (19)	179 (36)
Developed as a result of			
less severe incident	260 (41)	51 (34)	209 (43)
severe incident	173 (27)	70 (47)	103 (21)
Duration of symptoms, n (%)			
0–3 months	129 (20)	41 (27)	88 (18)
4–6 months	181 (28)	24 (16)	157 (32)
7–12 months	135 (21)	31 (21)	104 (21)
13–24 months	94 (15)	20 (13)	74 (15)
Longer than 24 months	102 (16)	34 (23)	68 (14)
KOOS subscale scores, mean (SD)			
Pain	54.9 (18)	58.9 (20)	53.6 (18)
Symptoms	60.0 (19)	60.6 (19)	59.8 (18)
ADL	63.7 (19)	69.8 (20)	61.8 (19)
Sport/Rec	26.3 (22)	31.1 (23)	24.9 (21)
QOL	41.6 (15)	40.2 (16)	42.0 (15)
Type of surgery, no. (%)			
Resection	600 (94)	118 (79)	482 (98.)
Repair	33 (5.1)	24 (16)	9 (1.8)
Both	8 (1.2)	8 (5.3)	0 (0.0)

KOOS: The Knee injury and Osteoarthritis Outcome Score with scores ranging from 0 to 100 (worst to best scale);

ADL: function in daily living;

Sport/Rec: function in sport and recreation;

QOL: quality of life.

### All patients (n = 641; n = 565 at 12 months’ follow-up)

**Figure 2.**
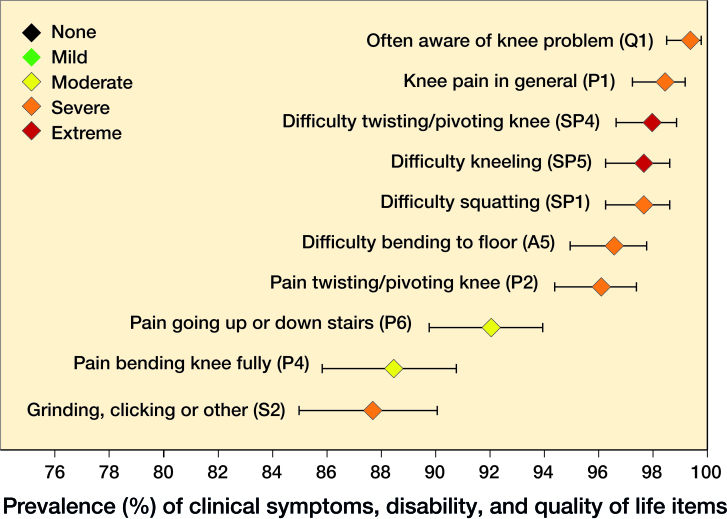
Prevalence (95% CI) and severity of the 5 most common clinical symptoms and five most common limitations and quality-of-life items in patients with a meniscal tear considered eligible for meniscal surgery (n = 641). Severity (color of the data points) is the most prevalent of the 5 levels of severity on the Knee Injury and Osteoarthritis Outcome Score (KOOS).

Increased awareness of the knee problem, knee pain in general, and difficulty twisting/pivoting the knee were the most prevalent symptoms and limitations. Severities of symptoms were mostly moderate to severe while severities of limitations were mostly severe to extreme for the most common symptoms and limitations (Tables 2 and 3, Figure 2 and Supplementary Tables 4 and 5).

**Table 2.** Prevalence and severity of the 5 most common clinical symptoms in the full group and the subgroups at baseline^a^

	All	≤ 40 years	> 40 years
	(n = 641)	(n = 150)	(n = 491)
Prevalence, n (%) [95% CI]			
S2. Grinding, clicking or other	562 (88) [85–90]	135 (90) [84–94]	427 (87) [84–90]^b^
S7. Stiffness later in the day	546 (85) [82–88]^b^	114 (76) [69–82]^b^	432 (88) [85–91]
P1. Knee pain in general	631 (98) [97–99]	147 (98) [95–99]	484 (99) [97–99]
P2. Pain twisting/pivoting knee	616 (96) [94–97]	141 (94) [89–97]	475 (97) [95–98]
P4. Pain bending knee fully	567 (89) [86–91]	131 (87) [81–92]	436 (89) [86–91]
P6. Pain going up or down stairs	590 (92) [90–94]	131 (87) [81–92]	459 (93) [91–95]
Severity^c^ (1/2/3/4), %			
S2. Grinding, clicking or other	12/31/42/15	12/26/43/19	12/26/43/19
S7. Stiffness later in the day	34/43/22/1	43/33/22/2	31/46/22/1
P1. Knee pain in general	5/11/65/19	8/20/53/19	4/9/69/18
P2. Pain twisting/pivoting knee	14/31/43/12	18/28/38/16	13/32/44/11
P4. Pain bending knee fully	24/33/31/11	25/31/26/18	24/34/33/9
P6. Pain going up or down stairs	24/36/32/9	34/34/21/11	21/36/35/8

95% CI =95% confidence intervals.

a Letters and numbers in front of each variable refer to item identification from the Knee Injury and Osteo-­arthritis Outcome Score (KOOS).

b Not in top 5 for the group, only included as comparator for the other groups.

c Severity: ranging from 1 (best) to 4 (worst) is the KOOS response categories for each individual item.

**Table 3.** Prevalence and severity of the 5 most common functional limitations and quality-of-life items in the full group and the subgroups at baseline^a^

	All	≤ 40 years	> 40 years
	(n = 641)	(n = 150)	(n = 491)
Prevalence, n (%) [95% CI]			
A5. Difficulty bending to floor	619 (97) [95–98]	144 (96) [92–98]^d^	475 (97) [95–98]
SP1. Difficulty squatting	626 (98) [96–99]	148 (99) [96–100]	478 (97) [96–98]
SP4. Difficulty twisting/pivoting knee	628 (98) [97–99]	144 (96) [92–98]^d^	484 (99) [97–99]
SP5. Difficulty kneeling	626 (98) [96–99]	145 (97) [93–99]	481 (98) [96–99]
Q1. Often aware of knee problem	637 (99) [98–100]	149 (99) [97–100]	488 (99) [98–100]
Q3. Lack of knee confidence	611 (95) [93–97]^b^	146 (97) [94–99]	465 (94) [92–96]^b^
Severity^c^ (1/2/3/4), %			
A5. Difficulty bending to floor	16/28/39/16	17/35/33/15	15/27/41/17
SP1. Difficulty squatting	8/18/37/37	11/18/36/34	8/17/37/38
SP2. Difficulty running	6/14/38/41	10/17/34/39	5/13/40/42
SP4. Difficulty twisting/pivoting knee	8/14/36/42	10/17/35/37	7/13/37/43
SP5. Difficulty kneeling	9/19/35/38	13/20/34/32	8/18/35/40
Q1. Often aware of knee problem	1/4/63/32	3/9/64/25	1/3/63/34
Q3. Lack of knee confidence	18/33/40/9	14/27/47/12	19/34/38/8

95% CI =95% confidence intervals.

a Letters and numbers in front of each variable refer to item identification from the Knee Injury and Osteo-­arthritis Outcome Score (KOOS).

b Not in top 5 for the group, only included as comparator for the other groups.

c Severity: ranging from 1 (best) to 4 (worst) is the KOOS response categories for each individual item.

d The prevalence was the same for these items, so both were included resulting in the young group having 6 most prevalent items.

**Figure 3.**
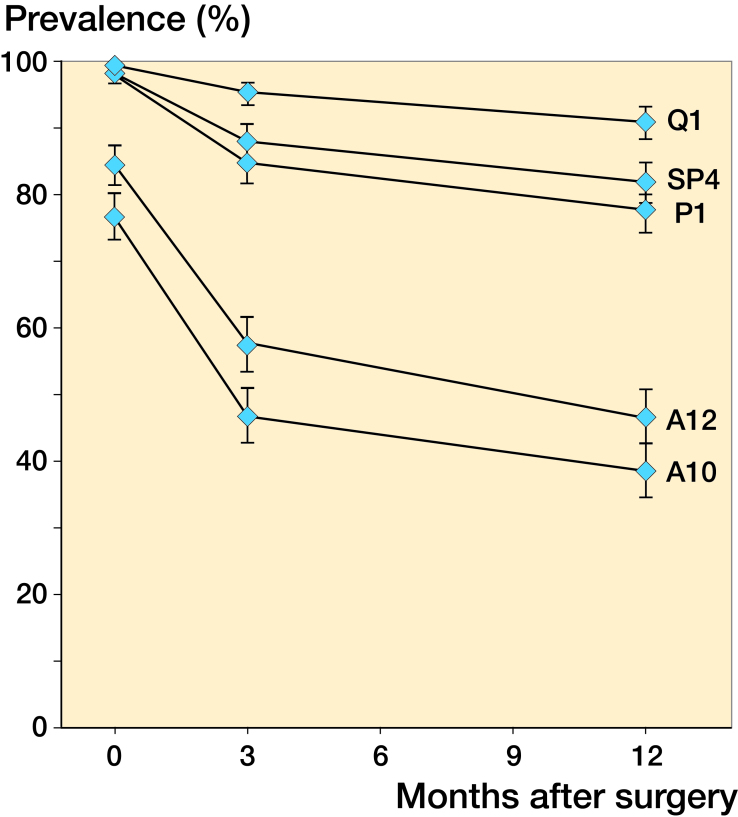
Prevalence (95% CI) of the 5 self-reported clinical symptoms and limitations and quality-of-life items mostly improved at 12 months after arthroscopic meniscal surgery at baseline, 3, and 12 months in patients with a meniscal tear considered eligible for meniscal surgery (n = 557). Letters and numbers in front of each variable refer to item identification from KOOS. Q1: Often aware of knee problem; SP4: Difficulty twisting/pivoting; P1: Knee pain in general; A12: Difficulty lying in bed; and A10: Difficulty rising from bed.

12 months after meniscal surgery, knee pain in general and awareness of the knee problem had improved most, while knee instability (actually worsened) and general knee difficulties had improved least (Table 6, Figure 3 and Supplementary Table 7).

**Table 6.** Outcome from baseline to 12 months for the 5 clinical symptoms and limitations and quality-of-life items mostly improved after arthroscopic surgery in the full group and for patients of 40 years of age or younger and patients older than 40 years of age^a^

	All (n = 565)				≤ 40 years (n = 121)				> 40 years (n = 444)			
	Base-line	12months	Diff.		Base-line	12months	Diff.		Base-line	12months	Diff.	
Severity^b^	n	n	p-value	Effect size	n	n	p-value	Effect size	n	n	p-value	Effect size
P1. Knee pain in general												
0	10	124	< 0.001	–0.47	3	20	< 0.001	–0.44	7	104	< 0.001	–0.48
1	29	132			11	33			18	99		
2	58	113			20	28			38	85		
3	371	161			68	31			303	130		
4	97	35			19	9			78	26		
P6. Pain going up and down stairs												
0	46	191	< 0.001	–0.42^c^	17	46	< 0.001	–0.30^c^	29	145	< 0.001	–0.45
1	125	158			38	32			87	126		
2	185	134			37	26			148	108		
3	169	71			22	13			147	58		
4	40	11			7	4			33	7		
A10. Difficulty rising from bed												
0	131	347	< 0.001	–0.44	51	89	< 0.001	–0.39^c^	80	258	< 0.001	–0.46
1	204	138			46	25			158	113		
2	162	59			19	7			143	52		
3	58	16			4	0			54	16		
4	10	5			1	0			9	5		
A12. Difficulty lying in bed (turning over, maintaining knee position)												
0	89	302	< 0.001	–0.44	36	74	< 0.001	–0.34^c^	53	228	< 0.001	–0.47
1	152	137			36	27			116	110		
2	183	79			32	11			151	68		
3	118	35			14	8			104	27		
4	23	12			3	1			20	11		
SP2. Difficulty running												
0	23	106	< 0.001	–0.41^c^	8	31	< 0.001	–0.43	15	75	< 0.001	–0.41^c^
1	34	117			12	28			22	89		
2	79	113			21	25			58	88		
3	208	133			37	21			171	112		
4	221	96			43	16			178	80		
SP4. Difficulty twisting/pivoting knee												
0	10	101	< 0.001	–0.43	4	27	< 0.001	–0.41	6	74	< 0.001	–0.44^c^
1	43	122			13	27			30	95		
2	85	121			25	29			60	92		
3	198	123			39	22			159	101		
4	229	98			40	16			189	82		
Q1. Often aware of knee problem												
0	3	51	< 0.001	–0.45	1	9	< 0.001	–0.40	2	42	< 0.001	–0.46
1	7	109			4	30			3	79		
2	21	105			8	24			13	81		
3	358	228			82	42			276	186		
4	176	72			26	16			150	56		
Q3. Lack of knee confidence												
0	25	108	< 0.001	–0.39^c^	3	19	< 0.001	–0.39	22	89	< 0.001	–0.39^c^
1	94	206			14	35			80	171		
2	183	120			36	33			147	87		
3	216	116			56	30			160	86		
4	47	15			12	4			35	11		

a Letters and numbers in front of each variable refer to item identification from the Knee Injury and Osteoarthritis Outcome Score (KOOS).

b Severity: ranging from 0 (best) to 5 (worst) or 0 (best) to 4 (worst) is the response categories for each individual item.

The comparison of the paired data was done using Wilcoxon’s signed-rank test.

Effect sizes were calculated by the formula r = Z/√N_observations_.

All comparisons between baseline and 12 months’ follow-up were significant (p-value <0.05).

c Not in top 5 for the group, only included as comparator for the other groups.

### Patients aged 40 years or younger (n = 150; n = 121 at 12 months’ follow-up)

Increased awareness of the knee problem, difficulties squatting, and knee pain in general were the most prevalent symptoms and limitations. Severities of symptoms were mostly moderate to severe while severities of limitations were mostly severe to extreme for the most common symptoms and limitations (Tables 2 and 3 and Supplementary Tables 4 and 5).

12 months after meniscal surgery, knee pain in general and difficulty running had improved most, knee instability (no difference between baseline and 12 months follow-up) and general knee difficulties had improved least (Table 6 and Supplementary Table 7).

### Patients aged more than 40 years (n = 491; n = 444 at 12 months’ follow-up)

Increased awareness of the knee problem, knee pain in general, and difficulty twisting/pivoting the knee were the most prevalent symptoms and limitations. Severities of symptoms were mostly moderate to severe while severities of limitations were mostly severe to extreme for the most common symptoms and limitations (Tables 2 and 3 and Supplementary Tables 4 and 5).

12 months after meniscal surgery, knee pain in general and difficulty lying in bed (turning over and maintaining knee position) had improved most, while knee instability (actually worsened) and general knee difficulties had improved least (Table 6 and Supplementary Table 7).

## Discussion

In this exploratory study we found that patient-reported grinding and clicking with knee movement, knee pain in general, and pain when twisting and bending the knee and going up and down stairs were the most common clinical symptoms experienced by patients about to undergo surgery for a meniscal tear. Difficulty bending to the floor, squatting, twisting, and kneeling, and awareness of the knee problem were the most common functional limitations and quality-of-life problems. These symptoms and functional limitations were present in 88–99% of all patients in the cohort and mostly of moderate to extreme severity. Furthermore, we found that patient-reported knee pain in general and knee awareness had improved most at 1 year after meniscal surgery, while knee instability and general knee difficulties improved least. Some differences did exist in subgroups of patients older or younger than 40 years of age.

### Prevalence and severity of symptoms

Meniscal injury is a common problem in the general population (Peat et al. 2014). However, detailed information on the most common self-reported symptoms, functional limitations, and quality-of-life problems, their severity, and improvements after treatment in patients with a meniscal tear is sparse despite its importance to guide the clinical discussion of symptoms, treatments, and patient expectations. A recent cross-sectional study of baseline data from 2 randomized controlled trials (RCT) of 199 middle-aged patients with a meniscal tear eligible for meniscal surgery found that knee pain in general, pain when twisting/pivoting the knee, when bending the knee fully, and when going up and down stairs, and lack of knee confidence were the 5 most common self-reported symptoms, functional limitations, and quality-of-life problems, all with at least moderate severity (Hare et al. 2017). In general, our results confirm these findings in a larger, prospective cohort that typically are more generalizable to the population undergoing meniscal surgery than participants recruited for RCTs with strict eligibility criteria (Bellomo and Bagshaw 2006). Furthermore, the clinical symptoms found to be most prevalent in our study are consistent with the meniscal symptoms that clinicians typically query patients about when diagnosing a meniscal tear (Niu et al. 2011), highlighting the relevance of asking these questions in clinical practice. However, many of these symptoms are also typical for patients with early knee osteoarthritis (Hare et al. 2017).

Niu et al. (2011) found that a Meniscal Symptom Index consisting of the combination of 4 symptoms (localized pain, clicking, catching, and giving way) were able to identify 76% of patients with symptoms consistent with a meniscal tear. As our cohort did not include patients without a meniscal tear, we were not able to assess the diagnostic value of a combination of symptoms to identify patients with a meniscal tear. However, a systematic review has shown that the accuracy of most commonly applied clinical tests of a meniscal tear is poor (Smith et al. 2015). Our findings may be used to develop and test a combined approach of clinical tests, medical history and self-reported symptoms for clinical diagnosing of a meniscal tear.

Interestingly, we found some subgroup differences in the most common clinical symptoms and functional limitations and quality-of-life problems. Knee stiffness after resting was only among the most prevalent symptoms in patients above 40 years of age (88%). Knee stiffness is a typical symptom of knee osteoarthritis (OA) (Zhang et al. 2010) and a meniscal tear in middle-aged and elderly people has been suggested to be a signifying feature of incipient OA (Englund et al. 2012). This corresponds well to the previous finding that a large proportion of patients in the present cohort have early or more established OA (Pihl et al. 2017). Lack of knee confidence was prevalent in the full group (95%), but only among the 5 most common functional limitations and quality-of-life problems in patients of 40 years or younger (97%). Worse knee confidence has previously been found to predict functional decline in people with or at increased risk of having knee OA (Colbert et al. 2012). Furthermore, it has been suggested to be an important part of a downward spiral with worse knee confidence decreasing the level of activity, potentially leading to worse pain again affecting knee confidence (Skou et al. 2014). This highlights the need to address knee confidence along with other symptoms in the treatment of patients with a meniscal tear.

### Changes in symptoms 1 year after meniscal surgery

Systematic reviews and meta-analyses of randomized controlled trials have failed to show superior effect of arthroscopic surgery compared with placebo surgery or in addition to exercise for middle-aged and older patients with degenerative meniscal tears (Khan et al. 2014, Thorlund et al. 2015, Brignardello-Petersen et al. 2017). Self-reported mechanical symptoms (i.e., the sensation of catching and/or locking) are typically considered an important indication for surgery (Stuart and Lubowitz 2006, Jevsevar et al. 2014, Krych et al. 2014). However, a recent study in patients with degenerative meniscal tears found no added benefit of surgery over that of placebo surgery in patients with preoperative mechanical symptoms (Sihvonen et al. 2018). Furthermore, the proportion of patients with preoperative mechanical symptoms satisfied with their knee status and reporting improvements at 1 year after arthroscopic surgery is lower compared with those without preoperative mechanical symptoms (Sihvonen et al. 2016b). In our study, mechanical symptoms (S2 and S3 on KOOS) only improved to a small extent 1 year following meniscal surgery. To some extent, this supports the findings from a secondary analysis of an RCT demonstrating that meniscal surgery has no added benefit over sham surgery in relieving knee catching or occasional locking (Sihvonen et al. 2016a).

Interestingly, although awareness of knee problem, difficulty twisting/pivoting knee, and knee pain in general were among the self-reported clinical symptoms and limitations and quality-of-life items that had improved most at 1 year after meniscal surgery, 77–91% of the patients still had the symptoms, suggesting that although patients can expect substantial improvements at 1 year after surgery, most will not be symptom free.

We found some differences in the subgroups of patients older or younger than 40 years, including smaller improvements in knee joint stiffness in the morning and in pain at night in the younger subgroup, potentially explained by differences in the severity of the symptoms at baseline in the different subgroups.

Based on data from the same cohort as used in this study, Thorlund et al. (2017) found a statistically larger, but clinically irrelevant, improvement in patient-reported outcomes following meniscal surgery in patients with a degenerative meniscal tear compared with patients who had a traumatic tear. However, evidence from RCTs supporting these findings is lacking. 2 ongoing trials will help shed light on the effects from meniscal surgery in a younger population, typically with a non-degenerative meniscal tear: 1 Dutch trial comparing arthroscopic resection and rehabilitation for a traumatic tear in adults aged 18–45 years of age (identifier www.trialregister.nl no. 17454) and 1 Danish trial of meniscal surgery versus exercise and education in patients of 40 or younger with a meniscal tear (Skou et al. 2017).

Our results provide guidance to clinicians and patients in terms of which symptoms patients can expect will improve most, and which symptoms will improve the least at 1 year after meniscal surgery and whether the patient can expect the individual symptom to disappear or be relieved.

### Limitations

Previous studies have suggested that the majority of the treatment effect from various treatments of knee pain is attributable to placebo or contextual factors (Bannuru et al. 2015, Zou et al. 2016). As our study is limited by the lack of a control group, the specific effect sizes found following meniscal surgery can only be used to compare changes in the different self-reported symptoms, functional limitations, and quality-of-life problems and not as a measure of the effects of surgery per se. Furthermore, the exploratory nature of this study precludes any firm conclusions.

155 patients did not reply to the baseline questionnaire before surgery. However, this was mainly because patients did not have sufficient time to respond, as waiting time for surgery was very short. The age of patients not responding was a bit lower (46 years of age vs. 49), while the gender distribution was similar (43% females vs. 44%). In general, demographics with regard to sex and age for patients included in this study are similar to what has previously been reported for patients undergoing meniscal surgery in Denmark (Thorlund et al. 2014), thus we consider the external validity of the results from the cohort to be high.

In summary, in patients undergoing meniscal surgery, grinding and clicking with knee movement, knee pain in general, and pain when twisting and bending the knee and going up and down stairs of mostly moderate to severe severity are common clinical symptoms. Difficulties bending to the floor, squatting, twisting, and kneeling, and awareness of the knee problem of mostly severe to extreme severity are common functional limitations and quality-of-life problems. At 1 year after surgery, moderate improvements were observed in some symptoms and functional limitations (knee pain in general and knee awareness improved most) and small to no improvement in others (knee instability and general knee difficulties improved least). Some differences did exist in the age-based subgroups, including worsened knee instability at 1 year after meniscal surgery in patients older than 40 years of age. Although the observational nature of our study precludes conclusions on the effects of surgery, the findings from our study can be used in the clinical discussion of symptoms, treatments, and patient expectations for patients with a meniscal tear.

### Supplementary data

Supplementary Tables 4, 5 and 7 are available as supplementary data in the online version of this article, http://dx.doi.org/10.1080/17453674.2018.1447281

JBT, KP, and STS conceived and designed the study. NN and UJ participated in patient recruitment and data collection and gave feedback on the study design. STS conducted the analysis. STS drafted the first version of the manuscript. All authors helped in revising the manuscript.

The authors would like to acknowledge the study funder, all participating patients, and orthopedic surgeons, nurses, and secretaries at the Department of Orthopedics and Traumatology at Odense University Hospital (Odense and Svendborg) and Department of Orthopedics at Lillebaelt Hospital (Kolding and Vejle) for their assistance with patient recruitment and data collection. STS is supported by the Danish Council for Independent Research (DFF - 6110-00045) and the Lundbeck Foundation.

*Acta* thanks Martin Englund and Rudolf Poolman for help with peer review of this study.

## Supplementary Material

IORT_A_1447281_SUPP.PDF

## References

[C1] Bannuru R R, McAlindon T E, Sullivan M C, Wong J B, Kent D M, Schmid C H. Effectiveness and implications of alternative placebo treatments: a systematic review and network meta-analysis of osteoarthritis trials. Ann Intern Med 2015; 163(5): 365–72. 26215539 10.7326/M15-0623

[C2] Bellomo R, Bagshaw S M. Evidence-based medicine: classifying the evidence from clinical trials—the need to consider other dimensions Crit Care 2006; 10(5): 232. 17029653 10.1186/cc5045 PMC1751050

[C3] Bergkvist D, Dahlberg L E, Neuman P, Englund M. Knee arthroscopies: who gets them, what does the radiologist report, what does the surgeon find? An evaluation from southern Sweden. Acta Orthop 2016; 87(1): 12–16. 26012547 10.3109/17453674.2015.1055179 PMC4940584

[C4] Brignardello-Petersen R, Guyatt G H, Buchbinder R, Poolman R W, Schandelmaier S, Chang Y, Sadeghirad B, Evaniew N, Vandvik P O. Knee arthroscopy versus conservative management in patients with degenerative knee disease: a systematic review. BMJ Open 2017; 7(5): e016114. 10.1136/bmjopen-2017-016114 PMC5541494 28495819

[C5] Colbert C J, Song J, Dunlop D, Chmiel J S, Hayes K W, Cahue S, Moisio K C, Chang A H, Sharma L. Knee confidence as it relates to physical function outcome in persons with or at high risk of knee osteoarthritis in the osteoarthritis initiative. Arthritis Rheum 2012; 64(5): 1437–46. 22135125 10.1002/art.33505 PMC3319513

[C6] Collins N J, Prinsen C A, Christensen R, Bartels E M, Terwee C B, Roos E M. Knee injury and Osteoarthritis Outcome Score (KOOS): systematic review and meta-analysis of measurement properties. Osteoarthritis Cartilage 2016; 24(8): 1317–29. 10.1016/j.joca.2016.03.010 27012756

[C7] Dawson J, Fitzpatrick R, Murray D, Carr A. Questionnaire on the perceptions of patients about total knee replacement. J Bone Joint Surg Br 1998; 80(1): 63–9. 9460955 10.1302/0301-620x.80b1.7859

[C8] Englund M, Guermazi A, Gale D, Hunter D J, Aliabadi P, Clancy M, Felson D T. Incidental meniscal findings on knee MRI in middle-aged and elderly persons. N Engl J Med 2008; 359(11): 1108–15. 18784100 10.1056/NEJMoa0800777 PMC2897006

[C9] Englund M, Roemer F W, Hayashi D, Crema M D, Guermazi A. Meniscus pathology, osteoarthritis and the treatment controversy. Nature Rev Rheumatol 2012; 8(7): 412–19. 22614907 10.1038/nrrheum.2012.69

[C10] Hare K B, Lohmander L S, Kise N J, Risberg M A, Roos E M. Middle-aged patients with an MRI-verified medial meniscal tear report symptoms commonly associated with knee osteoarthritis: a cross sectional study of 199 patients. Acta Orthop 2017; 88(6): 664–9. 28787249 10.1080/17453674.2017.1360985 PMC5694812

[C11] Herrlin S, Hallander M, Wange P, Weidenhielm L, Werner S. Arthroscopic or conservative treatment of degenerative medial meniscal tears: a prospective randomised trial. Knee Surg Sports Traumatol Arthrosc 2007; 15(4): 393–401. 17216272 10.1007/s00167-006-0243-2

[C12] Jevsevar D S, Yates A J Jr, Sanders J O. Arthroscopic partial meniscectomy for degenerative meniscal tear. N Engl J Med 2014; 370(13): 1260. 10.1056/NEJMc1401128 24670176

[C13] Katz J N, Brophy R H, Chaisson C E, de Chaves L, Cole B J, Dahm D L, Donnell-Fink L A, Guermazi A, Haas A K, Jones M H, Levy B A, Mandl L A, Martin S D, Marx R G, Miniaci A, Matava M J, Palmisano J, Reinke E K, Richardson B E, Rome B N, Safran-Norton C E, Skoniecki D J, Solomon D H, Smith M V, Spindler K P, Stuart M J, Wright J, Wright R W, Losina E. Surgery versus physical therapy for a meniscal tear and osteoarthritis. N Engl J Med 2013; 368(18): 1675–84. 23506518 10.1056/NEJMoa1301408 PMC3690119

[C14] Khan M, Evaniew N, Bedi A, Ayeni O R, Bhandari M. Arthroscopic surgery for degenerative tears of the meniscus: a systematic review and meta-analysis. CMAJ 2014; 186(14): 1057–64. 25157057 10.1503/cmaj.140433 PMC4188648

[C15] Krych A J, Carey J L, Marx R G, Dahm D L, Sennett B J, Stuart M J, Levy B A. Does arthroscopic knee surgery work? Arthroscopy 2014; 30(5): 544–5. 24642108 10.1016/j.arthro.2014.02.012

[C16] Lyman S, Oh L S, Reinhardt K R, Mandl L A, Katz J N, Levy B A, Marx R G. Surgical decision making for arthroscopic partial meniscectomy in patients aged over 40 years. Arthroscopy 2012; 28(4): 492–501.e491. 22264828 10.1016/j.arthro.2011.09.004

[C17] Niu N N, Losina E, Martin S D, Wright J, Solomon D H, Katz J N. Development and preliminary validation of a meniscal symptom index. Arthritis Care Res(Hoboken) 2011; 63(2): 208–15. 20862684 10.1002/acr.20354 PMC3025302

[C18] Peat G, Bergknut C, Frobell R, Joud A, Englund M. Population-wide incidence estimates for soft tissue knee injuries presenting to healthcare in southern Sweden: data from the Skane Healthcare Register. Arthritis Res Ther 2014; 16(4): R162. 25082600 10.1186/ar4678 PMC4262192

[C19] Pihl K, Englund M, Lohmander L S, Jorgensen U, Nissen N, Schjerning J, Thorlund J B. Signs of knee osteoarthritis common in 620 patients undergoing arthroscopic surgery for meniscal tear. Acta Orthop 2017; 88(1): 90–5. 27798972 10.1080/17453674.2016.1253329 PMC5251270

[C20] Poehling G G, Ruch D S, Chabon S J. The landscape of meniscal injuries. Clin Sports Med 1990; 9(3): 539–49. 2379242

[C21] Roos E M, Roos H P, Lohmander L S, Ekdahl C, Beynnon B D. Knee Injury and Osteoarthritis Outcome Score (KOOS): development of a self-administered outcome measure. J Orthop Sports Phys Ther 1998; 28(2): 88–96. 9699158 10.2519/jospt.1998.28.2.88

[C22] Siemieniuk R A C, Harris I A, Agoritsas T, Poolman R W, Brignardello-Petersen R, Van de Velde S, Buchbinder R, Englund M, Lytvyn L, Quinlan C, Helsingen L, Knutsen G, Olsen N R, Macdonald H, Hailey L, Wilson H M, Lydiatt A, Kristiansen A. Arthroscopic surgery for degenerative knee arthritis and meniscal tears: a clinical practice guideline. BMJ 2017; 357: j1982. 28490431 10.1136/bmj.j1982 PMC5426368

[C23] Sihvonen R, Englund M, Turkiewicz A. Jarvinen T L. Mechanical symptoms and arthroscopic partial meniscectomy in patients with degenerative meniscus tear: a secondary analysis of a randomized trial. Ann Intern Med 2016a; 164(7): 449–55. 26856620 10.7326/M15-0899

[C24] Sihvonen R, Englund M, Turkiewicz A. Jarvinen TL. Mechanical symptoms as an indication for knee arthroscopy in patients with degenerative meniscus tear: a prospective cohort study Osteoarthritis Cartilage 2016b; 24(8): 1367–75. 27038490 10.1016/j.joca.2016.03.013

[C25] Sihvonen R, Paavola M, Malmivaara A, Itala A, Joukainen A, Nurmi H, Kalske J, Ikonen A, Jarvela T, Jarvinen T A, Kanto K, Karhunen J, Knifsund J, Kroger H, Kaariainen T, Lehtinen J, Nyrhinen J, Paloneva J, Paivaniemi O, Raivio M, Sahlman J, Sarvilinna R, Tukiainen S, Valimaki V V, Aarimaa V, Toivonen P, Jarvinen T L. Arthroscopic partial meniscectomy versus placebo surgery for a degenerative meniscus tear: a 2-year follow-up of the randomised controlled trial. Ann Rheum Dis 2018; 77(2): 188–95. 28522452 10.1136/annrheumdis-2017-211172 PMC5867417

[C26] Skou S T, Wrigley T V, Metcalf B R, Hinman R S, Bennell K L. Association of knee confidence with pain, knee instability, muscle strength, dynamic varus–valgus joint motion in knee osteoarthritis. Arthritis Care Res 2014; 66(5): 695–701. 10.1002/acr.22208 24127243

[C27] Skou S T, Lind M, Hölmich P, Jensen H P, Jensen C, Afzal M, Jørgensen U, Thorlund J B. Study protocol for a randomised controlled trial of meniscal surgery compared with exercise and patient education for treatment of meniscal tears in young adults. BMJ Open 2017; 7(8): e017436. 10.1136/bmjopen-2017-017436 PMC5724132 28827270

[C28] Smith B E, Thacker D, Crewesmith A, Hall M. Special tests for assessing meniscal tears within the knee: a systematic review and meta-analysis. Evidence Based Med 2015; 20(3): 88–97. 10.1136/ebmed-2014-110160 25724195

[C29] Stuart M J, Lubowitz J H. What, if any, are the indications for arthroscopic debridement of the osteoarthritic knee? Arthroscopy 2006; 22(3): 238–9. 16523583 10.1016/j.arthro.2006.01.008

[C30] Thorlund J B, Christensen R, Nissen N, Jorgensen U, Schjerning J, Porneki J C, Englund M, Lohmander L S. Knee Arthroscopy Cohort Southern Denmark (KACS): protocol for a prospective cohort study. BMJ Open 2013; 3(10): e003399. 10.1136/bmjopen-2013-003399 PMC3808767 24127057

[C31] Thorlund J B, Hare K B, Lohmander L S. Large increase in arthroscopic meniscus surgery in the middle-aged and older population in Denmark from 2000 to 2011. Acta Orthop 2014; 85(3): 287–92. 24800623 10.3109/17453674.2014.919558 PMC4062797

[C32] Thorlund J B, Juhl C B, Roos E M, Lohmander L S. Arthroscopic surgery for degenerative knee: systematic review and meta-analysis of benefits and harms BMJ (Clin Res ed.) 2015; 350: h2747. 10.1136/bmj.h2747 PMC4469973 26080045

[C33] Thorlund J B, Englund M, Christensen R, Nissen N, Pihl K, Jorgensen U, Schjerning J, Lohmander L S. Patient reported outcomes in patients undergoing arthroscopic partial meniscectomy for traumatic or degenerative meniscal tears: comparative prospective cohort study. BMJ 2017; 356: j356. 28153861 10.1136/bmj.j356 PMC5421436

[C34] Yan R, Wang H, Yang Z, Ji Z H, Guo Y M. Predicted probability of meniscus tears: comparing history and physical examination with MRI. Swiss Med Wkly 2011; 141: w13314. 22180191 10.4414/smw.2011.13314

[C35] Zhang W, Doherty M, Peat G, Bierma-Zeinstra M A, Arden N K, Bresnihan B, Herrero-Beaumont G, Kirschner S, Leeb B F, Lohmander L S, Mazieres B, Pavelka K, Punzi L, So A K, Tuncer T, Watt I, Bijlsma J W. EULAR evidence-based recommendations for the diagnosis of knee osteoarthritis. Ann Rheum Dis 2010; 69(3): 483–9. 19762361 10.1136/ard.2009.113100

[C36] Zou K, Wong J, Abdullah N, Chen X, Smith T, Doherty M, Zhang W. Examination of overall treatment effect and the proportion attributable to contextual effect in osteoarthritis: meta-analysis of randomised controlled trials. Ann Rheum Dis 2016; 75(11): 7. 10.1136/annrheumdis-2015-208387 PMC5099197 26882927

