# Supplementary material for: Patient-reported symptoms and changes up to 1 year after meniscal surgery: An observational cohort study of 641 adult patients with a meniscal tear
Source: Acta Orthop. 2018 Mar 5;89(3):336–44. doi: 10.1080/17453674.2018.1447281 (PMC6055776; doi:10.1080/17453674.2018.1447281)
Supplement: IORT_A_1447281_SUPP.PDF [file IORT_A_1447281_SM0408.pdf]

Supplementary data

Supplementary Table 4. Prevalence of all clinical symptoms and disability and quaity-of-life items in the full group and the subgroups [a]

Factor	All (n = 641) n	%	95% CI	≤ 40 years (n = 150) n	%	95% CI	> 40 years (n = 491) n	%	95% CI
In the last month, have you felt that your knee was unstable or about to buckle?	502	78.3	75.0–81.4	127	84.7	78.3–89.7	375	76.4	72.5–80.0
S1. Do you have swelling in your knee?	513	80.0	76.8–83.0	113	75.3	68.0–81.7	400	81.5	77.9–84.7
S2. Do you feel grinding, hear clicking or any other type of noise when your knee moves?	562	87.7	85.0–90.0	135	90.0	84.4–94.0	427	87.0	83.8–89.7
S3. Does your knee catch or hang up when moving?	340	53.0	49.2–56.9	89	59.3	51.4–67.0	251	51.1	46.7–55.5
S4. Can you straighten your knee fully?	292	45.6	41.7–49.4	82	54.7	46.7–62.5	210	42.8	38.4–47.2
S5. Can you bend your knee fully?	457	71.3	67.7–74.7	110	73.3	65.9–79.9	347	70.7	66.5–74.6
S6. How severe is your knee joint stiffness after first waking in the morning?	518	80.8	77.6–83.7	106	70.7	63.0–77.5	412	83.9	80.5–87.0
S7. How severe is your knee stiffness after sitting, lying, or resting later in the day?	546	85.2	82.3–87.8	114	76.0	68.7–82.3	432	88.0	84.9–90.6
P1. How often do you experience knee pain?	631	98.4	97.3–99.2	147	98.0	94.8–99.4	484	98.6	97.2–99.4
P2. Twisting/pivoting on your knee	616	96.1	94.4–97.4	141	94.0	89.3–97.0	475	96.7	94.9–98.0
P3. Straightening knee fully	455	71.0	67.4–74.4	98	65.3	57.5–72.6	357	72.7	68.6–76.5
P4. Bending knee fully	567	88.5	85.8–90.8	131	87.3	81.3–91.9	436	88.8	85.8–91.4
P5. Walking on flat surface	483	75.4	71.9–78.6	102	68.0	60.2–75.1	381	77.6	73.8–81.1
P6. Going up or down stairs	590	92.0	89.8–94.0	131	87.3	81.3–91.9	459	93.5	91.0–95.4
P7. At night while in bed	443	69.1	65.5–72.6	78	52.0	44.0–59.9	365	74.3	70.3–78.1
P8. Sitting or lying	501	78.2	74.8–81.2	100	66.7	58.9–73.8	401	81.7	78.1–84.9
P9. Standing upright	538	83.9	80.9–86.6	122	81.3	74.5–86.9	416	84.7	81.3–87.7
A1. Descending stairs	567	88.5	85.8–90.8	123	82.0	75.3–87.5	444	90.4	87.6–92.8
A2. Ascending stairs	577	90.0	87.5–92.2	126	84.0	77.5–89.2	451	91.9	89.2–94.0
A3. Rising from sitting	557	86.9	84.1–89.3	111	74.0	66.6–80.5	446	90.8	88.0–93.1
A4. Standing	465	72.5	69.0–75.9	102	68.0	60.2–75.1	363	73.9	69.9–77.7
A5. Bending to floor/pick up an object	619	96.6	94.9–97.8	144	96.0	91.9–98.3	475	96.7	94.9–98.0
A6. Walking on flat surface	472	73.6	70.1–76.9	97	64.7	56.8–72.0	375	76.4	72.5–80.0
A7. Getting in/out of car	541	84.4	81.4–87.1	105	70.0	62.3–76.9	436	88.8	85.8–91.4
A8. Going shopping	459	71.6	68.0–75.0	84	56.0	48.0–63.8	375	76.4	72.5–80.0
A9. Putting on socks/stockings	435	67.9	64.2–71.4	86	57.3	49.3–65.0	349	71.1	67.0–75.0
A10. Rising from bed	488	76.1	72.7–79.3	89	59.3	51.4–67.0	399	81.3	77.6–84.5
A11. Taking off socks/stockings	438	68.3	64.7–71.8	90	60.0	52.0–67.6	348	70.9	66.7–74.8
A12. Lying in bed (turning over, maintaining knee position)	536	83.6	80.6–86.3	107	71.3	63.7–78.1	429	87.4	84.2–90.1
A13. Getting in/out of bath	344	53.7	49.8–57.5	68	45.3	37.5–53.3	276	56.2	51.8–60.6
A14. Sitting	432	67.4	63.7–70.9	91	60.7	52.7–68.2	341	69.5	65.3–73.4
A15. Getting on/off toilet	483	75.4	71.9–78.6	96	64.0	56.1–71.4	387	78.8	75.0–82.3
A16. Heavy domestic duties (moving heavy boxes, scrubbing floors, etc.)	570	88.9	86.3–91.2	122	81.3	74.5–86.9	448	91.2	88.5–93.5
A17. Light domestic duties (cooking, dusting, etc.)	430	67.1	63.4–70.6	89	59.3	51.4–67.0	341	69.5	65.3–73.4
SP1. Squatting	626	97.7	96.3–98.6	148	98.7	95.8–99.7	478	97.4	95.6–98.5
SP2. Running	613	95.6	93.8–97.0	140	93.3	88.5–96.5	473	96.3	94.4–97.7
SP3. Jumping	600	93.6	91.5–95.3	133	88.7	82.9–93.0	467	95.1	92.9–96.8
SP4. Twisting/pivoting on your injured knee	628	98.0	96.7–98.9	144	96.0	91.9–98.3	484	98.6	97.2–99.4
SP5. Kneeling	626	97.7	96.3–98.6	145	96.7	92.8–98.7	481	98.0	96.4–98.9
Q1. How often are you aware of your knee problem?	637	99.4	98.5–99.8	149	99.3	96.9–99.9	488	99.4	98.4–99.8
Q2. Have you modified your lifestyle to avoid potentially damaging activities	601	93.8	91.7–95.4	138	92.0	86.8–95.6	463	94.3	92.0–96.1
Q3. How much are you troubled with lack of confidence in your knee?	611	95.3	93.5–96.8	146	97.3	93.8–99.1	465	94.7	92.5–96.4
Q4. In general, how much difficulty do you have with your knee?	509	79.4	76.1–82.4	123	82.0	75.3–87.5	386	78.6	74.8–82.1

[a] Letters and numbers in front of each variable refer to item identification from the Knee Injury and Osteoarthritis Outcome Score (KOOS); 95% CI = 95% Confidence Intervals

 Acta Orthopaedica 2018; 89 (DOI: 10.1080/17453674.2018.1447281)

Supplementary Table 5. Severity of all clinical symptoms and disability and quality-of-life items in the full group and the subgroups*

Factor Severity [b]	All (n = 641) n	%	≤ 40 years (n = 150) n	%	> 40 yrars (n = 491) n	%
In the last month, have you felt that your knee was unstable or about to buckle?						
1	238	47.4	57	44.9	181	48.3
2	113	22.5	26	20.5	87	23.2
3	47	9.4	16	12.6	31	8.3
4	13	2.6	4	3.1	9	2.4
5	91	18.1	24	18.9	67	17.9
S1. Do you have swelling in your knee?						
1	109	21.2	28	24.8	81	20.3
2	175	34.1	39	34.5	136	34.0
3	137	26.7	31	27.4	106	26.5
4	92	17.9	15	13.3	77	19.3
S2. Do you feel grinding, hear clicking or any other type of noise when your knee moves?						
1	68	12.1	16	11.9	52	12.2
2	174	31.0	35	25.9	139	32.6
3	234	41.6	58	43.0	176	41.2
4	86	15.3	26	19.3	60	14.1
S3. Does your knee catch or hang up when moving?						
1	102	30.0	18	20.2	84	33.5
2	135	39.7	35	39.3	100	39.8
3	87	25.6	30	33.7	57	22.7
4	16	4.7	6	6.7	10	4.0
S4. Can you straighten your knee fully?						
1	133	45.5	41	50.0	92	43.8
2	70	24.0	18	22.0	52	24.8
3	32	11.0	7	8.5	25	11.9
4	57	19.5	16	19.5	41	19.5
S5. Can you bend your knee fully?						
1	127	27.8	30	27.3	97	28.0
2	112	24.5	23	20.9	89	25.6
3	83	18.2	24	21.8	59	17.0
4	135	29.5	33	30.0	102	29.4
S6. How severe is your knee joint stiffness after first waking in the morning?						
1	186	35.9	56	52.8	130	31.6
2	192	37.1	28	26.4	164	39.8
3	123	23.7	19	17.9	104	25.2
4	17	3.3	3	2.8	14	3.4
S7. How severe is your knee stiffness after sitting, lying, or resting later in the day?						
1	184	33.7	49	43.0	135	31.3
2	235	43.0	38	33.3	197	45.6
3	119	21.8	25	21.9	94	21.8
4	8	1.5	2	1.8	6	1.4
P1. How often do you experience knee pain?						
1	31	4.9	12	8.2	19	3.9
2	71	11.3	29	19.7	42	8.7
3	412	65.3	78	53.1	334	69.0
4	117	18.5	28	19.0	89	18.4
P2. Twisting/pivoting on your knee						
1	86	14.0	26	18.4	60	12.6
2	192	31.2	40	28.4	152	32.0
3	263	42.7	53	37.6	210	44.2
4	75	12.2	22	15.6	53	11.2
P3. Straightening knee fully						
1	170	37.4	41	41.8	129	36.1
2	171	37.6	30	30.6	141	39.5
3	95	20.9	23	23.5	72	20.2
4	19	4.2	4	4.1	15	4.2
P4. Bending knee fully						
1	137	24.2	33	25.2	104	23.9
2	189	33.3	40	30.5	149	34.2
3	178	31.4	34	26.0	144	33.0
4	63	11.1	24	18.3	39	8.9
P5. Walking on flat surface						
1	217	44.9	47	46.1	170	44.6
2	212	43.9	41	40.2	171	44.9
3	47	9.7	9	8.8	38	10.0
4	7	1.4	5	4.9	2	0.5
P6. Going up or down stairs						
1	140	23.7	45	34.4	95	20.7
2	211	35.8	45	34.4	166	36.2
3	186	31.5	27	20.6	159	34.6
4	53	9.0	14	10.7	39	8.5
P7. At night while in bed						
1	163	36.8	36	46.2	127	34.8
2	159	35.9	25	32.1	134	36.7
3	102	23.0	13	16.7	89	24.4
4	19	4.3	4	5.1	15	4.1
P8. Sitting or lying						
1	235	46.9	63	63.0	172	42.9
2	199	39.7	24	24.0	175	43.6
3	59	11.8	11	11.0	48	12.0
4	8	1.6	2	2.0	6	1.5
P9. Standing upright						
1	212	39.4	56	45.9	156	37.5
2	241	44.8	48	39.3	193	46.4
3	74	13.8	14	11.5	60	14.4
4	11	2.0	4	3.3	7	1.7
A1. Descending stairs						
1	165	29.1	45	36.6	120	27.0
2	200	35.3	43	35.0	157	35.4
3	158	27.9	26	21.1	132	29.7
4	44	7.8	9	7.3	35	7.9
A2. Ascending stairs						
1	174	30.2	48	38.1	126	27.9
2	207	35.9	40	31.7	167	37.0
3	152	26.3	27	21.4	125	27.7
4	44	7.6	11	8.7	33	7.3
A3. Rising from sitting						
1	157	28.2	39	35.1	118	26.5
2	217	39.0	43	38.7	174	39.0
3	163	29.3	26	23.4	137	30.7
4	20	3.6	3	2.7	17	3.8
A4. Standing						
1	242	52.0	60	58.8	182	50.1
2	159	34.2	27	26.5	132	36.4
3	55	11.8	12	11.8	43	11.8
4	9	1.9	3	2.9	6	1.7
A5. Bending to floor/pick up an object						
1	97	15.7	24	16.7	73	15.4
2	176	28.4	50	34.7	126	26.5
3	244	39.4	48	33.3	196	41.3
4	102	16.5	22	15.3	80	16.8
A6. Walking on flat surface						
1	238	50.4	49	50.5	189	50.4
2	192	40.7	37	38.1	155	41.3
3	38	8.1	10	10.3	28	7.5
4	4	0.8	1	1.0	3	0.8
A7. Getting in/out of car						
1	202	37.3	46	43.8	156	35.8
2	214	39.6	40	38.1	174	39.9
3	109	20.1	16	15.2	93	21.3
4	16	3.0	3	2.9	13	3.0
A8. Going shopping						
1	218	47.5	42	50.0	176	46.9
2	175	38.1	34	40.5	141	37.6
3	58	12.6	6	7.1	52	13.9
4	8	1.7	2	2.4	6	1.6

A9. Putting on socks/stockings						
1	204	46.9	46	53.5	158	45.3
2	156	35.9	31	36.0	125	35.8
3	62	14.3	8	9.3	54	15.5
4	13	3.0	1	1.2	12	3.4
A10. Rising from bed						
1	228	46.7	52	58.4	176	44.1
2	183	37.5	31	34.8	152	38.1
3	65	13.3	5	5.6	60	15.0
4	12	2.5	1	1.1	11	2.8
A11. Taking off socks/stockings						
1	218	49.8	54	60.0	164	47.1
2	160	36.5	24	26.7	136	39.1
3	53	12.1	10	11.1	43	12.4
4	7	1.6	2	2.2	5	1.4
A12. Lying in bed (turning over, maintaining knee position)						
1	175	32.6	46	43.0	129	30.1
2	201	37.5	38	35.5	163	38.0
3	133	24.8	18	16.8	115	26.8
4	27	5.0	5	4.7	22	5.1
A13. Getting in/out of bath						
1	181	52.6	44	64.7	137	49.6
2	133	38.7	19	27.9	114	41.3
3	25	7.3	4	5.9	21	7.6
4	5	1.5	1	1.5	4	1.4
A14. Sitting						
1	242	56.0	60	65.9	182	53.4
2	155	35.9	25	27.5	130	38.1
3	30	6.9	5	5.5	25	7.3
4	5	1.2	1	1.1	4	1.2
A15. Getting on/off toilet						
1	238	49.3	55	57.3	183	47.3
2	176	36.4	31	32.3	145	37.5
3	56	11.6	8	8.3	48	12.4
4	13	2.7	2	2.1	11	2.8
A16. Heavy domestic duties (moving heavy boxes, scrubbing floors, etc.)						
1	165	28.9	43	35.2	122	27.2
2	206	36.1	47	38.5	159	35.5
3	151	26.5	22	18.0	129	28.8
4	48	8.4	10	8.2	38	8.5
A17. Light domestic duties (cooking, dusting, etc.)						
1	209	48.6	48	53.9	161	47.2
2	174	40.5	32	36.0	142	41.6
3	37	8.6	6	6.7	31	9.1
4	10	2.3	3	3.4	7	2.1
SP1. Squatting						
1	53	8.5	16	10.8	37	7.7
2	110	17.6	27	18.2	83	17.4
3	232	37.1	54	36.5	178	37.2
4	231	36.9	51	34.5	180	37.7
SP2. Running						
1	38	6.2	14	10.0	24	5.1
2	87	14.2	24	17.1	63	13.3
3	234	38.2	47	33.6	187	39.5
4	254	41.4	55	39.3	199	42.1
SP3. Jumping						
1	65	10.8	21	15.8	44	9.4
2	109	18.2	30	22.6	79	16.9
3	211	35.2	46	34.6	165	35.3
4	215	35.8	36	27.1	179	38.3
SP4. Twisting/pivoting on your injured knee						
1	48	7.6	15	10.4	33	6.8
2	89	14.2	25	17.4	64	13.2
3	228	36.3	51	35.4	177	36.6
4	263	41.9	53	36.8	210	43.4
SP5. Kneeling						
1	57	9.1	19	13.1	38	7.9
2	116	18.5	29	20.0	87	18.1
3	216	34.5	50	34.5	166	34.5
4	237	37.9	47	32.4	190	39.5
Q1. How often are you aware of your knee problem?						
1	8	1.3	4	2.7	4	0.8
2	27	4.2	13	8.7	14	2.9
3	400	62.8	95	63.8	305	62.5
4	202	31.7	37	24.8	165	33.8
Q2. Have you modified your life style to avoid potentially damaging activities						
1	130	21.6	27	19.6	103	22.2
2	177	29.5	36	26.1	141	30.5
3	244	40.6	56	40.6	188	40.6
4	50	8.3	19	13.8	31	6.7
Q3. How much are you troubled with lack of confidence in your knee?						
1	110	18.0	21	14.4	89	19.1
2	200	32.7	40	27.4	160	34.4
3	245	40.1	68	46.6	177	38.1
4	56	9.2	17	11.6	39	8.4
Q4. In general, how much difficulty do you have with your knee?						
1	148	29.1	30	24.4	118	30.6
2	213	41.8	59	48.0	154	39.9
3	131	25.7	28	22.8	103	26.7
4	17	3.3	6	4.9	11	2.8

[a] Letters and numbers in front of each variable refer to item identification from the Knee Injury and Osteoarthritis Outcome Score (KOOS).

[b] Severity: ranging from 1 (best) to 5 (worst) or 1 (best) to 4 (worst) is the response categories for each individual item.

Supplementary Table 7. Outcome from baseline to 12 months for all clinical symptoms and disability and quality-of-life items in the full group and for patients of 40 years of age or younger [a]

	All (n = 565)				≤ 40 years (n = 121)				> 40 years (n = 444)			
Item<br>Severity [b]	Baseline n	12 months n	p-value	Diff. Effect size	Baseline n	12 months n	p-value	Diff. Effect size	Baseline n	12 months n	p-value	Diff. Effect size
In the last month, have you felt that your knee was unstable or about to buckle? [c]												
0	125	162	0.001 [b]	0.10	19	29	0.11	0.10	106	133	0.005 [b]	0.09
1	210	148			46	31			164	117		
2	95	66			21	11			74	55		
3	39	22			11	8			28	14		
4	12	9			3	5			9	4		
5	83	157			21	37			62	120		
S1. Do you have swelling in your knee?												
0	115	269	< 0.001 [b]	–0.35	32	64	< 0.001 [b]	–0.27	83	205	< 0.001 [b]	–0.38
1	95	103			24	14			71	89		
2	156	102			32	25			124	77		
3	119	49			22	11			97	38		
4	80	42			11	7			69	35		
S2. Do you feel grinding. hear clicking or any other type of noise when your knee moves?												
0	69	132	< 0.001 [b]	–0.23	13	25	0.001 [b]	–0.21	56	107	< 0.001 [b]	–0.24
1	62	98			16	21			46	77		
2	156	161			27	35			129	126		
3	204	123			47	27			157	96		
4	74	51			18	13			56	38		
S3. Does your knee catch or hang up when moving?												
0	273	406	< 0.001 [b]	–0.29	50	76	< 0.001 [b]	–0.30	223	330	< 0.001 [b]	–0.28
1	89	86			17	25			72	61		
2	116	41			28	9			88	32		
3	76	23			23	9			53	14		
4	11	9			3	2			8	7		
S4. Can you straighten your knee fully?												
0	315	413	< 0.001 [b]	–0.20	59	86	< 0.001 [b]	–0.26	256	327	< 0.001 [b]	–0.19
1	114	93			31	24			83	69		
2	62	18			15	3			47	15		
3	27	14			5	4			22	10		
4	47	27			11	4			36	23		
S5. Can you bend your knee fully?												
0	163	273	< 0.001 [b]	–0.26	33	52	0.001 [b]	–0.21	130	221	< 0.001 [b]	–0.27
1	115	144			28	35			87	109		
2	100	36			17	5			83	31		
3	72	47			21	11			51	36		
4	115	65			22	18			93	47		
S6. How severe is your knee joint stiffness after first waking in the morning?												
0	105	191	< 0.001 [b]	–0.29	36	38	0.16	–0.09	69	153	< 0.001 [b]	–0.34
1	165	205			46	53			119	152		
2	176	109			27	23			149	86		
3	105	55			9	7			96	48		
4	14	5			3	0			11	5		
S7. How severe is your knee stiffness after sitting, lying, or resting later in the day?												
0	86	196	< 0.001 [b]	–0.32	31	46	0.002 [b]	–0.20	55	150	< 0.001 [b]	–0.34
1	160	203			39	43			121	160		
2	209	109			32	20			177	89		
3	103	49			17	10			86	39		
4	7	8			2	2			5	6		
P1. How often do you experience knee pain?												
0	10	124	< 0.001 [b]	–0.47	3	20	< 0.001 [b]	–0.44	7	104	< 0.001 [b]	–0.48
1	29	132			11	33			18	99		
2	58	113			20	28			38	85		
3	371	161			68	31			303	130		
4	97	35			19	9			78	26		
P2. Twisting/pivoting on your knee												
0	23	139	< 0.001 [b]	–0.43	8	28	< 0.001 [b]	–0.35	15	111	< 0.0012	–0.45
1	77	171			21	37			56	134		
2	169	136			34	31			135	105		
3	236	92			44	17			192	75		
4	60	27			14	8			46	19		

P3. Straightening knee fully												
0	168	344	< 0.001 [b]	–0.33	47	76	< 0.001 [b]	–0.26	121	268	< 0.001 [b]	–0.35
1	149	111			32	23			117	88		
2	153	76			24	15			129	61		
3	81	26			16	5			65	21		
4	14	8			2	2			12	6		
P4. Bending knee fully												
0	68	206	< 0.001 [b]	–0.36	18	42	< 0.001 [b]	–0.30	50	164	< 0.001 [b]	–0.38
1	119	146			28	31			91	115		
2	172	127			34	28			138	99		
3	158	57			26	13			132	44		
4	48	29			15	7			33	22		
P5. Walking on flat surface												
0	141	353	< 0.001 [b]	–0.38	41	77	< 0.001 [b]	–0.31	100	276	< 0.001 [b]	–0.39
1	189	127			36	25			153	102		
2	194	62			37	15			157	47		
3	37	19			5	4			32	15		
4	4	4			2	0			2	4		
P6. Going up or down stairs												
0	46	191	< 0.001 [b]	–0.42	17	46	< 0.001 [b]	–0.30	29	145	< 0.001 [b]	–0.45
1	125	158			38	32			87	126		
2	185	134			37	26			148	108		
3	169	71			22	13			147	58		
4	40	11			7	4			33	7		
P7. At night while in bed												
0	172	380	< 0.001 [b]	–0.38	60	89	< 0.001 [b]	–0.23	112	291	< 0.001 [b]	–0.42
1	148	95			31	19			117	76		
2	143	55			21	6			122	49		
3	89	26			7	5			82	21		
4	13	9			2	2			11	7		
P8. Sitting or lying												
0	126	345	< 0.001 [b]	–0.40	46	73	< 0.001 [b]	–0.26	80	272	< 0.001 [b]	–0.44
1	207	136			48	35			159	101		
2	181	63			19	9			162	54		
3	46	18			6	4			40	14		
4	5	3			2	0			3	3		
P9. Standing upright												
0	93	281	< 0.001	–0.40	24	65	< 0.001	–0.35	69	216	< 0.001 [b]	–0.41
1	189	165			47	31			142	134		
2	211	88			37	16			174	72		
3	63	26			11	8			52	18		
4	9	5			2	1			7	4		
A1. Descending stairs												
0	66	225	< 0.001 [b]	–0.43	23	56	< 0.001	–0.39	43	169	< 0.001 [b]	–0.44
1	145	173			38	37			107	136		
2	177	99			35	19			142	80		
3	140	55			19	6			121	49		
4	37	13			6	3			31	10		
A2. Ascending stairs												
0	57	221	< 0.001 [b]	–0.42	22	51	< 0.0012	–0.37	35	170	< 0.001 [b]	–0.43
1	156	165			39	39			117	126		
2	182	108			33	21			149	87		
3	135	61			21	7			114	54		
4	35	10			6	3			29	7		
A3. Rising from sitting												
0	76	227	< 0.001 [b]	–0.41	35	56	< 0.001 [b]	–0.27	41	171	< 0.001 [b]	–0.45
1	139	182			33	35			106	147		
2	189	101			33	22			156	79		
3	147	46			19	8			128	38		
4	14	9			1	0			13	9		
A4. Standing												
0	153	317	< 0.001 [b]	–0.33	39	68	< 0.0012	–0.29	114	249	< 0.001 [b]	–0.35
1	214	145			49	35			165	110		
2	144	82			23	16			121	66		
3	46	17			7	1			39	16		
4	8	4			3	1			5	3		
A5. Bending to floor/to pick up an object												
0	20	128	< 0.001 [b]	–0.41	5	27	< 0.001 [b]	–0.34	15	101	< 0.001 [b]	–0.43
1	84	155			20	30			64	125		
2	156	136			42	36			114	100		
3	224	105			41	21			183	84		
4	81	41			13	7			68	34		

A6. Walking on flat surface												
0	153	359	< 0.0012	–0.39	44	80	< 0.001 [b]	–0.31	109	279	< 0.001 [b]	–0.41
1	210	137			41	26			169	111		
2	169	57			31	14			138	43		
3	30	10			4	1			26	9		
4	3	2			1	0			2	2		
A7. Getting in/out of car												
0	86	287	< 0.001 [b]	–0.43	37	74	< 0.001 [b]	–0.35	49	213	< 0.001 [b]	–0.45
1	182	151			40	28			142	123		
2	190	92			32	14			158	78		
3	96	31			10	5			86	26		
4	11	4			2	0			9	4		
A8. Going shopping												
0	161	372	< 0.001 [b]	–0.41	53	85	< 0.001 [b]	–0.31	108	287	< 0.001 [b]	–0.43
1	191	121			37	23			154	98		
2	156	49			24	9			132	40		
3	51	19			5	3			46	16		
4	6	4			2	1			4	3		
A9. Putting on socks/stockings												
0	184	356	< 0.001 [b]	–0.36	55	82	< 0.001 [b]	–0.29	129	274	< 0.001 [b]	–0.38
1	179	129			37	28			142	101		
2	139	61			25	8			114	53		
3	54	17			4	3			50	14		
4	9	2			0	0			9	2		
A10. Rising from bed												
0	131	347	< 0.001 [b]	–0.44	51	89	< 0.001 [b]	–0.39	80	258	< 0.001 [b]	–0.46
1	204	138			46	25			158	113		
2	162	59			19	7			143	52		
3	58	16			4	0			54	16		
4	10	5			1	0			9	5		
A11. Taking off socks/stockings												
0	183	362	< 0.001 [b]	–0.36	52	82	< 0.001 [b]	–0.27	131	280	< 0.001 [b]	–0.38
1	191	131			43	28			148	103		
2	139	54			19	6			120	48		
3	48	15			6	4			42	11		
4	4	3			1	1			3	2		
A12. Lying in bed (turning over, maintaining knee position)												
0	89	302	< 0.001 [b]	–0.44	36	74	< 0.001 [b]	–0.34	53	228	< 0.001 [b]	–0.47
1	152	137			36	27			116	110		
2	183	79			32	11			151	68		
3	118	35			14	8			104	27		
4	23	12			3	1			20	11		
A13. Getting in/out of bath												
0	266	415	< 0.001 [b]	–0.31	69	93	< 0.001 [b]	–0.24	197	322	< 0.001 [b]	–0.32
1	153	99			34	19			119	80		
2	119	39			14	7			105	32		
3	24	10			4	2			20	8		
4	3	2			0	0			3	2		
A14. Sitting												
0	190	382	< 0.001 [b]	–0.36	51	82	< 0.001 [b]	–0.28	139	300	< 0.001 [b]	–0.37
1	214	122			48	28			166	94		
2	132	45			17	9			115	36		
3	25	13			4	2			21	11		
4	4	3			1	0			3	3		
A15. Getting on/off toilet												
0	144	329	< 0.001 [b]	–0.37	48	77	< 0.001 [b]	–0.31	96	252	< 0.001 [b]	–0.39
1	206	148			43	30			163	118		
2	155	60			23	13			132	47		
3	51	23			6	1			45	22		
4	9	5			1	0			8	5		
A16. Heavy domestic duties (moving heavy boxes. scrubbing floors, etc.)												
0	61	223	< 0.001 [b]	–0.41	23	57	< 0.001 [b]	–0.34	38	166	< 0.001 [b]	–0.43
1	147	168			37	31			110	137		
2	178	97			38	20			140	77		
3	139	59			17	10			122	49		
4	40	18			6	3			34	15		
A17. Light domestic duties (cooking. dusting, etc.)												
0	189	370	< 0.001 [b]	–0.37	52	82	< 0.001 [b]	–0.32	137	288	< 0.001 [b]	–0.39
1	185	128			41	28			144	100		
2	151	55			21	9			130	46		
3	32	10			5	2			27	8		
4	8	2			2	0			6	2		

SP1. Squatting												
0	14	70	< 0.001 [b]	–0.38	2	20	< 0.001 [b]	–0.38	12	50	< 0.001 [b]	–0.38
1	46	149			13	32			33	117		
2	97	117			23	24			74	93		
3	210	119			46	24			164	95		
4	198	110			37	21			161	89		
SP2. Running												
0	23	106	< 0.001 [b]	–0.41	8	31	< 0.001	–0.43	15	75	< 0.001 [b]	–0.41
1	34	117			12	28			22	89		
2	79	113			21	25			58	88		
3	208	133			37	21			171	112		
4	221	96			43	16			178	80		
SP3. Jumping												
0	36	133	< 0.001 [b]	–0.39	15	39	< 0.001 [b]	–0.35	21	94	< 0.001 [b]	–0.40
1	58	115			17	25			41	90		
2	93	130			24	24			69	106		
3	190	100			37	21			153	79		
4	188	87			28	12			160	75		
SP4. Twisting/pivoting on your injured knee												
0	10	101	< 0.001 [b]	–0.43	4	27	< 0.001	–0.41	6	74	< 0.001 [b]	–0.44
1	43	122			13	27			30	95		
2	85	121			25	29			60	92		
3	198	123			39	22			159	101		
4	229	98			40	16			189	82		
SP5. Kneeling												
0	15	75	< 0.001 [b]	–0.35	5	23	< 0.001 [b]	–0.36	10	52	< 0.001 [b]	–0.35
1	51	116			16	30			35	86		
2	102	127			24	23			78	104		
3	194	130			42	26			152	104		
4	203	117			34	19			169	98		
Q1. How often are you aware of your knee problem?												
0	3	51	< 0.001 [b]	–0.45	1	9	< 0.001 [b]	–0.40	2	42	< 0.001 [b]	–0.46
1	7	109			4	30			3	79		
2	21	105			8	24			13	81		
3	358	228			82	42			276	186		
4	176	72			26	16			150	56		
Q2. Have you modified your lifestyle to avoid potentially damaging activities												
0	30	128	< 0.001 [b]	–0.37	7	20	< 0.001 [b]	–0.29	23	108	< 0.001 [b]	–0.39
1	111	209			23	42			88	167		
2	166	98			31	22			135	76		
3	216	111			48	31			168	80		
4	42	19			12	6			30	13		
Q3. How much are you troubled with lack of confidence in your knee?												
0	25	108	< 0.0012	–0.39	3	19	< 0.0012	–0.39	22	89	< 0.0012	–0.39
1	94	206			14	35			80	171		
2	183	120			36	33			147	87		
3	216	116			56	30			160	86		
4	47	15			12	4			35	11		
Q4. In general, how much difficulty do you have with your knee?												
0	118	88	0.005 [b]	–0.08	22	15	0.13	–0.10	96	73	0.005 [b]	–0.08
1	129	223			25	51			104	172		
2	194	178			52	42			142	136		
3	115	68			20	11			95	57		
4	9	8			2	2			7	6		

[a] Letters and numbers in front of each variable refer to item identification from the Knee Injury and Osteoarthritis Outcome Score (KOOS). Severity: ranging from 0 (best) to 5 (worst) or 0 (best) to 4 (worst) is the response categories for each individual item. The comparison of the paired data was done using Wilcoxon's signed-rank test. Effect sizes were calculated by the formula $r = Z/\sqrt{N_{observations}}$.
[b] Significant difference between baseline and 12 months' follow-up (p-value < 0.05).
[c] Data from one patient from the full group and the group older than 40 years of age was not available for this question at 12 months.
